# The Role of Heparanase and Sulfatases in the Modification of Heparan Sulfate Proteoglycans within the Tumor Microenvironment and Opportunities for Novel Cancer Therapeutics

**DOI:** 10.3389/fonc.2014.00195

**Published:** 2014-07-24

**Authors:** Edward Hammond, Ashwani Khurana, Viji Shridhar, Keith Dredge

**Affiliations:** ^1^Progen Pharmaceuticals Ltd., Brisbane, QLD, Australia; ^2^Department of Experimental Pathology, Mayo Clinic College of Medicine, Rochester, MN, USA

**Keywords:** heparanase, Sulf1, Sulf2, heparan sulfate, cancer therapy, tumor microenvironment targeting

## Abstract

Heparan sulfate proteoglycans (HSPGs) are an integral and dynamic part of normal tissue architecture at the cell surface and within the extracellular matrix. The modification of HSPGs in the tumor microenvironment is known to result not just in structural but also functional consequences, which significantly impact cancer progression. As substrates for the key enzymes sulfatases and heparanase, the modification of HSPGs is typically characterized by the degradation of heparan sulfate (HS) chains/sulfation patterns via the endo-6-*O*-sulfatases (Sulf1 and Sulf2) or by heparanase, an endo-glycosidase that cleaves the HS polymers releasing smaller fragments from HSPG complexes. Numerous studies have demonstrated how these enzymes actively influence cancer cell proliferation, signaling, invasion, and metastasis. The activity or expression of these enzymes has been reported to be modified in a variety of cancers. Such observations are consistent with the degradation of normal architecture and basement membranes, which are typically compromised in metastatic disease. Moreover, recent studies elucidating the requirements for these proteins in tumor initiation and progression exemplify their importance in the development and progression of cancer. Thus, as the influence of the tumor microenvironment in cancer progression becomes more apparent, the focus on targeting enzymes that degrade HSPGs highlights one approach to maintain normal tissue architecture, inhibit tumor progression, and block metastasis. This review discusses the role of these enzymes in the context of the tumor microenvironment and their promise as therapeutic targets for the treatment of cancer.

## Introduction

Heparan sulfate (HS) chains are an important component of the extracellular matrix (ECM) and they facilitate a number of important biological processes in health and disease. Incorporated into heparan sulfate proteoglycans (HSPG), they comprise a significant fraction of the complex and dynamic ECM and contribute to the biological roles of this medium. The functions of HSPG are varied: the maintenance of a 3D structural matrix for cell attachment; in certain cases the formation of barrier structures (basement membranes); the provision of a reservoir of biological signaling molecules; acting as a cofactor for signaling molecules binding to their receptors (1–3). These functions are modulated by proteins that are released into the ECM to modify its constituent molecules. Three important proteins within this category are heparanase (4–6), Sulf1, and Sulf2 (7, 8), which are released by a variety of cells to modify the structure of HS and thereby alter the function of the ECM.

This review will focus upon the roles of these three enzymes in modifying HS in the tumor microenvironment to facilitate processes that support or repress cancer growth and spread. The importance of heparanase, Sulf1, and Sulf2 will also be demonstrated by exploring their association with poor patient prognosis in clinical studies. Finally, the relevant therapeutic developments in the area will be summarized.

## Enzyme Properties

### Heparanase

There is only one heparanase gene known in mammals (HPSE in humans) and it expresses a 65 kDa precursor polypeptide that is enzymatically inactive. Proteolytic excision by cathepsin L of a 48 amino acid peptide yields an active heterodimer comprising 8 and 50 kDa subunits. Although the structures of precursor and active heparanase are unknown, it has been postulated that the excised linker sequence occludes the active site based on sequence analysis and that once removed the active site is revealed allowing catalysis to proceed (9). Sequence homology modeling has indicated that heparanase contains a TIM barrel fold, which incorporates the two HS-binding regions (residues 158–162 and 270–280) and the catalytic residues (Glu225 and Glu343) of the active site (10–14). The C terminus of the protein forms a discrete domain, which has non-catalytic properties and is involved in a heparanase-mediated signaling function that is distinct from its enzymatic activity (15, 16). This review will focus upon the enzymatic activity of heparanase and its role in regulating HS functioning in the ECM.

Catalytically, heparanase is an endo-β glucuronidase that cleaves HS chains at a limited number of sites within HS chains yielding relatively large degradation fragments [5–10 kDa or 10–20 sugar units (17)]. The exact substrate requirements for heparanase have not been clarified and recent studies suggest that it has alternating modes of catalysis (18), but it appears to favor trisaccharide sequences containing flanking glucosamine residues that are *N*- and 6-*O* sulfated (19). The pH optimum for heparanase catalysis peaks at 5 and the enzyme possesses little activity above 7 (20).

### Sulf1 and Sulf2

Sulf1 and Sulf2 are two closely related members of the sulfatase family (human genes SULF1 and SULF2). Unlike most other sulfatases, which are intracellular and function in the catabolism of sulfated molecules, Sulf1 and Sulf2 are extracellularly targeted and function by selectively removing 6-*O*-sulfate groups from glucosamine residues within HS polymers. Like heparanase, the Sulfs are also expressed as precursor polypeptides (125 kDa) and are processed by a furin-type protease to produce the mature heterodimer comprising 75 and 50 kDa subunits connected by disulfide linkages (21, 22). Although the active site is contained in the 75 kDa subunit, both subunits are required for activity. In contrast to heparanase, the pH optima for the Sulfs are 7–8 (23). Significantly, catalysis by either Sulf to remove 6-*O*-sulfates from HS chains would reduce the affinity that heparanase has for these portions of the glycosaminoglycan. It is not clear, however, whether the Sulfs play a role in modulating heparanase activity and, thus, regulating its function *in vivo*.

### Molecular processes in tumor microenvironment

**Figure 1:**
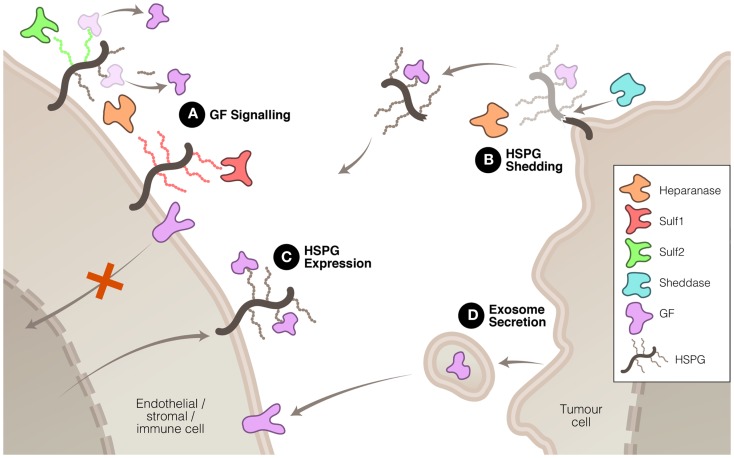
**Mechanisms whereby heparanase, Sulf1, and Sulf2 regulate HS function to promote or inhibit tumor growth and spread**. **(A)** HS modification by these three enzymes can have promoting or inhibiting effects on growth factor (GF) signaling. **(B)** Heparanase, in association with sheddases, can stimulate HSPG shedding, dispersing autocrine to paracrine signaling. **(C)** Heparanase and Sulf2 can up-regulate HSPG expression to promote GF signaling. And **(D)** heparanase can induce exosome secretion allowing tumor cell communication with neighboring cells.

Heparanase, Sulf1, and Sulf2 have been shown, by virtue of their enzymatic modification of the ECM, to affect the signaling of a number of proteins that are important drivers of tumor growth or progression. In some instances, the signaling function of heparanase may be involved in driving these changes, but it is accepted that heparanase cleavage of HS also plays a significant role in modulating signal transduction. These three enzymes accomplish this modulation in signaling in a number of ways (Figure 1). Firstly, heparanase and Sulf activity modifies the HS component of the ECM, thus altering the interactions between signaling molecules and their receptors. Secondly, heparanase is closely linked to the process of syndecan shedding, particularly syndecan-1, which can be important in changing the focus of a signaling stimulus, for example from autocrine to paracrine. Thirdly, heparanase and Sulf2 regulate expression of HSPG at the cell surface, thus promoting HS-dependant signaling. And fourthly, heparanase is involved in exosome formation by cancer cells, which has recently been shown to promote tumor progression by acting upon both cancerous and stromal cells.

### HS modification to modulate signaling

The modification of HS chains in the ECM to liberate stored signaling molecules in the tumor microenvironment is a mechanism that heparanase and the sulfatases have in common. There is, however, some diversity in the outcomes resulting from the modification of HS by these three enzymes. Heparanase cleavage of HS has been shown to increase the solubility of a variety of signaling molecules including VEGF (24) and FGF2 (25, 26) thus increasing their access to receptors and facilitating signal transduction. This property has linked heparanase with the promotion of a number of pro-tumor processes including angiogenesis, cell proliferation and invasion, inhibition of apoptosis, and metastasis.

In addition to its catalysis resulting in increased availability of signaling molecules, heparanase cleavage of HSPG can also facilitate the interaction of these molecules with their receptors. Moderate heparanase activity has been shown to potentiate FGF2 signaling (27). This effect appears to be due to the solubilization of HS chains by heparanase as demonstrated by the observation that heparanase-cleaved HS chains stimulated FGF2 activity in the absence of heparanase itself. Soluble HS may improve the likelihood of formation of the active FGF2–FGFR–HS ternary complex by easing the conformational constraints of this assembly. A contrasting mechanism was discovered with lacritin in which heparanase promotes the activity of this mitogenic protein by removing the HS chains from syndecan-1 leaving the deglycanated HSPG as the active receptor for lacritin (28).

Rather than having uniformly tumor-promoting properties, Sulf1 and Sulf2 have pro- and anti-tumor affects. Functionally, activity of both sulfatases is linked to angiogenesis, albeit in opposing manner. Despite their similar enzymatic activities, evidence suggests that Sulf1 and Sulf2 play opposing roles in the progression of tumors. For example, Sulf1 has been shown to attenuate the signaling of HS-binding growth factors such as FGF2 (29, 30), VEGF (30), amphiregulin (31), HB-EGF (29), and HGF (32, 33). Such effects are mediated by reduced affinity of these signaling proteins for vascular endothelial HS, thus reducing their concentration at the cell surface and minimizing interaction with their cognate receptors. Perhaps, the most studied example is the attenuation of FGF2 signaling by Sulf1. The removal of 6-*O*-sulfate groups by Sulf1 reduces formation of the active ternary FGF2–FGFR–HS complex and consequently blocks FGF2 signaling (34, 35). However, the regulatory functions of Sulf1 have added complexity because alternative splicing of this gene can generate isoforms, which have different effects on Wnt signaling (36).

In contrast to Sulf1, Sulf2 has been shown to mobilize and increase the signaling of FGF2 in HCC cells (37). Interestingly, it accomplishes this despite having the same 6-*O*-endosulfatase activity as Sulf1 and despite the observed FGF2 signaling remaining HS dependant (37). Sulf2 has shown similar promotion of signaling with several other HS-binding signaling proteins including VEGF (38), Wnt (39, 40), SDF1 (38), and GDNF (41). Apart from the opposing roles played by Sulf1 and Sulf2 in regulating tumor growth through mobilizing heparan binding growth factors, these endosulfatases are also involved in regulating apoptotic proteins enhancing the sensitivity to chemotherapeutic agents in ovarian cancer. Recently, it was shown that Sulf1 depletion in ovarian cancer cells resulted in marked decrease in pro-apoptotic protein such as Bim, thus promoting tumor growth (42). Although reduction in Bim levels were attributed to high ERK activities, it underscores the possibility that lower levels of apoptotic proteins will favor resistance to chemotherapeutic agents. Along these lines, it was demonstrated that genetic silencing of Sulf1 in ovarian cancer cells attenuated cisplatin induced cytotoxicity (43). Consistent with their opposing roles in growth factor signaling, Sulf2 expression attenuated cell death induction by MEK, JNK, and PI3K inhibitors in hepatocellular carcinoma cell lines. These data were further strengthened by the finding that Sulf2 expression levels were elevated in human hepatocellular carcinoma (44). Overall, it is conceivable to conclude that Sulf1 plays anti-tumor roles whereas Sulf2 plays tumor-promoting roles in several tumor types. The paradox of how two sulfatases with similar catalytic activities have different biological functions and outcomes in tumor types, remains to be solved.

### Syndecan shedding

The expression and mobilization of HSPG, such as syndecan, in the tumor and stroma microenvironment is associated with poor prognosis in a number of cancers. Expressed at the cell surface, these proteoglycans can be shed by the action of proteases referred to as sheddases. The protein core of the syndecan HSPG is cleaved by proteases, such as MMP-9, releasing it from attachment to the cell membrane. Although sheddases are responsible for releasing syndecan from the cell surface, heparanase promotes this process in two ways: firstly, by degrading HS chains, it increases the rate at which sheddases cleave the core protein (45) and secondly, heparanase can increase the expression of sheddases such as MMP-9 (46). Shed syndecan, because it commonly contains bound signaling proteins, facilitates the translocation of these signals from the expressing cell, which may be a tumor cell, to stromal cells, thus converting autocrine signaling into a paracrine signal (47–49). It is noteworthy that shed syndecan ectodomain, which can be detected in sampled plasma or serum, represents a potential biomarker for the malignancy status of cancer (50) and its response to a heparanase-targeting therapeutic.

### HS upregulation

Both heparanase and Sulf2 are capable of increasing expression of HSPG targeted to the cell surface and, consequently, increasing pro-tumor signaling by HS-binding growth factors. Sulf2 has been shown to activate the Wnt pathway via upregulating glypican 3 at the cell surface, which results in enhanced Wnt–Frz complex formation and increased Wnt-β-catenin signaling (37, 39, 51). Similarly, heparanase has been demonstrated to regulate the expression of syndecan-1 in multiple myeloma cells (52). Heparanase promotes the expression of this cell surface HSPG, which is involved in facilitating the transduction of a range of signaling pathways including FGF2 (53), Wnt (54), and HGF (55). Apart from exerting control over the total mass of HS, there is evidence to suggest that heparanase also affects the composition of the HS chains. Higher levels of heparanase expression correlated with increased *N*- and *O*-sulfation of HS chains which lead to potentiation of FGF1 and FGF2 signaling (56).

### Exosome secretion

Another mechanism, recently discovered, by which heparanase is able to promote tumor malignancy is via the stimulation of exosome secretion (57), although as yet Sulf1 and Sulf2 have not been shown to be involved in this process. Exosomes are lipid membrane bound extracellular vesicles that allow tumor cells to communicate with and co-opt neighboring cells to assist in modifying the tumor microenvironment and, thus, promote the tumor’s growth and spread (58–60). Heparanase enzymatic activity has been shown to promote the formation of exosomes by several types of cancer cells and, moreover, to affect the molecular composition of the exosomes (57). The resulting exosomes were potent stimulators of both tumor and endothelial cells (Figure 1). The significance of this process in cancer has yet to be established but, if confirmed, would highlight the diversity of heparanase functions in tumor promotion.

### Potent regulators of HS function

Heparanase, Sulf1, and Sulf2 drive a range of processes in the tumor microenvironment, many of which, in the case of heparanase and Sulf2 promote tumor malignancy, and some, in the case of Sulf1 inhibit malignancy. Despite their shared substrate and significance in cancer progression, it is important to note that the pH optima of heparanase (pH 5) and the Sulfs (pH 7–8) preclude them from being at their most active in the same microenvironment compartment, or at the same time. The low extracellular pH associated with tumor-driven hypoxia has the consequence of stimulating heparanase-mediated activation of pro-angiogenic growth factors leading to a replenishment of the tumor microenvironment (61–63). Interestingly, Sulf1 and Sulf2 show the opposite environmental regulation being suppressed during hypoxia (64, 65). This is unsurprising for Sulf1, given its largely antagonistic function compared to heparanase. Sulf2, however, has pro-angiogenic properties like heparanase but is, nonetheless, downregulated under conditions requiring new blood supply. This apparent paradox suggests a complicated regulatory interaction between heparanase and the Sulfs. One could speculate that the Sulfs, with their neutral pH optimum and fine interplay between pro- and anti-angiogenic functions, are the early regulators of HS remodeling to control angiogenesis but, if metabolic changes or tumor growth drive the compartment into hypoxia, then heparanase becomes the leading regulator.

## Cellular Processes

For some time, the involvement of heparanase in the metastatic extravasation of tumor cells and invasion of immune cells has been known. We will not cover in detail the work describing the importance of heparanase in these processes but will instead refer the interested reader to the excellent reviews that have already summarized this data (5, 6, 66). Rather, we will highlight one of the areas of research that has recently increased our understanding of heparanase functioning at the cellular level: namely its role in promoting epithelial–mesenchymal transition (EMT).

The transition of epithelial cells from their polarized state to non-polarized multipotent mesenchymal cells is an important process during metastasis. Recent studies examining EMT of kidney tubule epithelial cells into myofibroblasts show that heparanase is involved in driving this process by promoting FGF2 signaling (67). The mechanism whereby heparanase promotes this activity is not clear but it appears to involve enzymatic activity because the heparanase inhibitor sulodexide abrogates the effect (68). While these results were obtained using kidney tubule cells, it is intriguing to speculate that the heparanase-FGF2 axis plays an important role in cancer spread due to the well established connection between FGF2 and EMT in a variety of other situations, including tumor cells (69–72).

Given the role of FGF2 in heparanase-mediated EMT, it may be discovered that Sulfs also have roles in regulating EMT due to their interactions with this growth factor. In support of this, there is a recent finding that suppression of Sulf1, among other proteins, was associated with activation of EMT in HCC cells via increased signaling through the Akt and ERK pathways, which would be expected after Sulf1-mediated FGF2 inhibition was alleviated (73).

## Clinical Association Studies

Reports identifying an association of heparanase, Sulf1, and/or Sulf2 expression or protein levels from clinical samples have been discussed in extensive detail in many excellent reviews (7, 8, 66, 74–76). Nevertheless, Table 1 highlights several recent studies, which have identified an association of these degrading extracellular enzymes of HS with tumor progression, metastasis, and poor prognosis, and illustrates some of the scientific and clinical findings in a variety of cancer types.

**Table 1:** **Association of heparanase, Sulf1, and Sulf2 in solid tumors and hematologic malignancies**.

Enzyme	Cancer	Key scientific finding	Key clinical finding	Reference
Heparanase	PNET	HPSE mRNA ↑ by 40-fold in primary tumors and metastatic tumors compared to normal islets	HPSE mRNA was significantly upregulated in PNET patients with primary tumors (*P = 0.046, *n* = 25) and liver metastases (*P = 0.026, *n* = 15) compared to normal islet samples from normal islets (*n* = 4)	(78)
Heparanase	OMM	HPSE staining was positive in 81% of tumors (66 of 81 patients)	Median survival time and 5-year survival rate were 12 months and 7.0% in the high-HPSE group, 35 months and 36.4% in the low-HPSE group, 62 months and 53.3% in the no-HPSE group (*P* = 0.001)	(79)
Heparanase	Cervical	HPSE ↑ by 63% (38/60) patients by IHC	Significant correlation with tumor size and clinical stage	(80)
Heparanase	Ovarian	Median HPSE serum levels 2.77, 4.86 and 7.68 ng/mL in control, benign and malignant samples respectively	Increased serum HPSE in ovarian cancer patients with distant metastasis (*P* < 0.05) HPSE (in conjunction with Cathepsin L and MMP-9) elevation possibly useful in determining extent of metastasis before surgery	(81)
Heparanase	Oral SCC	HPSE ↑ in 41% (19/46)	Rate of HPSE expression closely related to tumor size, tumor stage, lymphatic metastasis, distant metastasis, pathological and histological stage	(82)
Heparanase	Lung*	Adenocarcinomas exhibited strong HPSE expression by IHC	Heparanase expression tended to correlate with tumor node metastasis (TNM) staging in non-small cell lung carcinoma	(83)
Heparanase	CCC	HPSE expression from 47 patient samples was significantly associated with PDGFRα expression but not its ligand PDGF	HPSE expression (mRNA) <35th percentile led to median OS 10.2 months versus HPSE >35th percentile lead to median OS 20.1 months	(84)
Heparanase	HNSCC	Strong HPSE expression was localized at the invasion front of the tumor and in disseminated tumor cells	Patients lacking HPSE-expressing cells (<5%) in their tumors had a prolonged DFS of 25.8 compared with patients with HPSE-positive tumors (>5%) with mean DFS of 8.2 months. HPSE also higher in lymph nodes	(85)
		Cellular HPSE expression was detected in 41 of 71 (58%) cases; in particular, UICC IV-stage tumors	Patients with high-level HPSE expression had prolonged overall survival (*P* = 0.029) and this was associated with low tumor cell proliferation which might outweigh HPSE-induced invasion and migration in late-stage tumors	(86)
Heparanase	Ewing’s sarcoma	49% (34/69) Of the cases were scored as low (+1) intensity while 51% (35/69) exhibited a strong (+2) staining intensity	HPSE staining was evident in all biopsies examined, exhibiting a high (+2) staining extent (i.e., >50% of the cells) in the majority (91%) of cases. Possible association to tumor size (*P* = 0.07), strong staining in 75% of those with large tumors (>10 cm)	(77)
	STS	High-HPSE expression in 29 (52.7%) primary tumors and 22 specimens (47.8%) in metastatic sites	HPSE expression not correlative with tumor aggressiveness, tumor recurrence or survival	(87)
Heparanase	AML	mRNA and/or protein expression of HPSE revealed low HPSE in ALL (and CLL, NHL) patients and a high expression level in MM and AML patients, versus healthy controls	HPSE mRNA expression was significantly increased in 14/15 patient samples and genotype frequency comparisons revealed a significant association with rs4364254 [chi2 (2d.f.) = 6.226, *P* = 0.044] in AML patients. HPSE gene mRNA expression was high in AML patients	(88, 89)
Sulf1	Gastric	Sulf1 expression ↑in tumor tissues (*P* = 0.0002) compared to normal mucosa	Multivariate analysis found Sulf1 is an independent prognostic (*P* = 0.01) and lymph node metastasis predictive factor (*P* = 0.0003) in large cohort of patients (450)	(90)
Sulf1	Gastric	Sulf1 protein expression ↓which is discordant with mRNA findings	Despite mRNA expression being ↑in 30% of samples, protein expression ↓in 70% (14/20) of tumor samples	(91)
Sulf2	GBM	Sulf2 may alter PDGFRα signaling/activation to promote tumorigenesis	Sulf2 expression ↑in proneural subtype of GBM (*n* = 173, *P* < 0.005) and ↑using IHC in 28 subtyped GBMs demonstrated that proneural and mesenchymal subtypes	(92)
Sulf2	MM	Sulf2 ↑in hyperdiploid group but ↓in groups of patients with Cyclin D1 or MAF translocations	Sulf2 expression in primary MM cells linked to poor prognosis in two independent large cohorts. Sulf2 was independently predictive for OS (*P* = 0.02)	(93)
Sulf2	OAC/OSCC	Sulf2 detected using IHC on 75 OAC patients and 25 OSCC patients	Majority of OAC and OSCC had Sulf2 staining. For every 10%, ↑in % tumor cells staining for Sulf2, the HR for death ↑by 13% (*P* = 0.03)	(94)
Sulf2	Various	Sulf2 increased (*P* = 0.001) compared to normal mucosa	Significant overexpression in uveal melanoma (=0.03), lung adenocarcinoma (*P* = 0.04) and colorectal carcinoma (*P* = 0.001) compared to low grade, low expressed and colon adenomas respectively	(93)

*AML, acute myeloid leukemia; CCC, cholangiocarcinoma; CLL, chronic lymphoblastic leukemia; DFS, disease-free survival; GBM, glioblastoma multiforme; HCC, hepatocellular carcinoma; HNSCC, head and neck squamous cell carcinoma; MM, multiple myeloma; NHL, non-Hodgkin’s lymphoma; OAC, esophageal adenocarcinoma; OMM, oral mucosal melanoma; OSCC, esophageal squamous cell carcinoma; PNET, pancreatic neuroendocrine tumors; SCC, squamous cell carcinoma; STS, soft tissue sarcoma*.

*37 Patients, there were 14 adenocarcinomas, 13 squamous cell carcinomas, 5 large cell carcinomas, and 5 small cell carcinomas.

It has been postulated that heparanase and HSPGs act synergistically within the tumor microenvironment to enhance tumor growth and the enzymatic versus non-enzymatic roles of heparanase in cancer have been discussed elsewhere (74). Although the active form of heparanase has been identified as a key player in multiple myeloma (52) and sarcoma (77), several other studies have linked the overexpression of the active form with aggressive primary tumor growth, invasion, and metastasis in certain cancer types. In contrast, Sulf1 has been found to be downregulated in a number of cancer types while Sulf2 is typically overexpressed in carcinomas but importantly, these may be differentially expressed depending on the stage of cancer and the level of hypoxia in the tumor microenvironment (7). This section highlights some of the prevalent cancer types, which have reported significant alterations in heparanase, Sulf1, and/or Sulf2 and how these changes in expression correlate with indices of cancer progression such as prognostic indicators, tumor development, metastasis, and survival.

## Solid Tumors

### Pancreatic cancer

Overexpression of heparanase in pancreatic adenocarcinomas (PDAC) was identified several years ago when investigators discovered that this may facilitate cancer cell invasion and enhancement of metastatic dissemination (95). Postoperative survival correlated inversely with heparanase expression by tumors reflected by a median survival of 34 and 17 month for heparanase negative and positive tumors, respectively (96). Using RT-PCR and Western Blotting techniques, Chen and colleagues demonstrated that the expression of HPSE was significantly associated with TNM grade and invasion to nerves or lymph nodes although did not reveal a significant difference between histological differentiation and the tumor size (97). Even serum heparanase was found to be significantly elevated in PDAC patients (especially in those with heparanase positive tumors by IHC) and treatment with a small cohort of patients (*n* = 11) reduced heparanase levels by 64% after 2 weeks of treatment with gemcitabine (98). More recently, pancreatic neuroendocrine tumors (PNET) have now also been identified as a cancer type in which positive heparanase expression was detected in patient tissue samples. Using tissue microarrays with samples from over 150 PNET patients, staining intensity of heparanase (by IHC) significantly correlated with tumor stage, higher tumor grade (as defined by tumor mitotic activity), and presence of distant metastasis (78).

Given the increasing understanding of the HS-binding protein interactome in pancreatic disease, enzymes involved in the degradation of proteoglycans such as HS may well play particularly important roles in neoplastic transformation of pancreatic cells. Although Sulf1 inhibits angiogenesis and tumorigenesis *in vivo* (30), a number of clinical studies have found that higher Sulf1 mRNA levels have been associated with tumor tissue compared to normal pancreas in relatively small cohorts of PDAC patients (99, 100). The finding that Sulf1 and Sulf2 can promote canonical Wnt signaling, a well described cascade in PDAC, suggests their overexpression is a contributory factor in relation to the growth and tumorigenicity of these pancreatic tumor cells (101). This appears to be in contrast to evidence that Sulfs block other pro-tumorigenic signaling pathways such as angiogenesis (29, 30, 33, 100), thus blocking tumor progression. However, the ability of Sulf1 to potentiate autocrine Wnt signaling in pancreatic cancer cells appears to be the key factor for tumors driven by this canonical signaling pathway (101). Taken together, heparanase, Sulf1, and Sulf2 are enzymes which appear, at least in pancreatic cancer, to be positive regulators of tumor development.

### Hepatocellular carcinoma

In patients undergoing hepatic resection, expression of heparanase mRNA was detected in 47% of HCCs and was significantly correlated with larger tumor size, presence of portal vein invasion, higher overall tumor invasiveness, and tumor microvessel density (MVD). Also, there was a direct correlation between the level of FGF2 protein and MVD in HCC tissue suggesting that heparanase enhances growth, invasion, and angiogenesis while FGF2 is a potent angiogenic factor for HCC (102). Interestingly, Sulf2 is known to increase FGF2 binding to HCC cells and upregulation of Sulf2 correlates with a worse prognosis in HCC patients (37). Chen and colleagues subsequently found a similar positive rate of increased expression of heparanase mRNA (48.5%) in HCC tumors compared to surrounding parenchymal tissue. However, the positive rate increased to 71.4% in patients with a higher tendency of metastasis or recurrence compared with 31.6% in the group with a low tendency of metastasis or recurrence. The positive rate for mRNA heparanase in patients with metastasis/recurrence during postoperative follow-up (78.6%, 11/14) was also significantly higher than that in those without metastatic recurrence (21.4%, 3/14), indicating heparanase may be one of the reliable markers for metastatic activity in HCC (103). A follow-up study revealed heparanase expression was increased in patients with metastasis and was dependent on tumor staging with expression levels lower in clinical TNM stages I and II than in III and IV (104). Serum heparanase levels have also been reported to be higher in patients with large tumors (>5 cm), advanced pTNM stage (III and IV), tumor capsule absence, and portal vein invasion (105).

Although Sulf2 is purported to exhibit an oncogenic effect in HCC (37), in contrast, Sulf1 has been identified as having a tumor suppressor effect in HCC (32, 106). However, in HCC tumor tissue, expression of Sulf1 is higher compared to adjacent benign tissues and approximately a third of HCCs express Sulf1 at high levels >1.5× the level in adjacent benign tissue (107). Moreover, nearly 40% of patients with high tumor Sulf1 expression have the hepatoblast phenotype of HCC, which has relatively poor survival (108) and those with mid Sulf1 expression had a better survival outcome possibly due to the complex interaction of Sulf1 with anti- and pro-tumorigenic signaling molecules, indicating a bimodal effect in HCC (107). In contrast, elevated Sulf2 is associated with a worse prognosis and a unimodal effect in HCC, causing activation of both tyrosine kinase and Wnt pathways (107). A different study also reported increased expression of Sulf2 in liver cancer specimens compared to normal tissue counterparts (93).

### Gastric cancers

Rates of positive expression for heparanase mRNA in gastric cancer tissues (31/63, 49%) compared with adjacent normal tissue (11/42, 26%) was first reported by Endo and colleagues in 2001 (109). A follow-up study confirmed 79.5% (35/44) positive expression in heparanase protein using immunohistochemistry and also reported significantly poorer prognosis than those without such expression (110). A number of subsequent studies confirmed the increase expression of mRNA or protein in gastric cancer correlated with invasion, metastasis, and/or poor survival outcomes (103, 111–115).

Higher expression of Sulf1 and Sulf2 compared to normal mucosa has been reported in gastric cancer, with the expression of Sulf1 significantly correlated with higher recurrence rates and worse overall survival in patients. Multivariate analysis revealed that Sulf1 is an independent prognostic factor and lymph node metastasis predictive factor in these patients (90). However, Sulf1 protein expression has also been reported as being down regulated in gastric cancer tissues, which is discordant with mRNA overexpression in tumors previously reported by the same laboratory (91), thus, demonstrating the complexity in associating Sulf expression/activity with clinicopathological settings. Laboratory data reports would suggest that Sulf1 inhibits cell proliferation and invasion in human gastric cells (116) and suppresses cell growth while down regulating the pro-tumorigenic Hedgehog signaling pathway in these cells (117).

### Head and neck cancers

Strong heparanase expression in primary tumors and lymph nodes was initially reported to be associated with prolonged disease-free progression in head and neck squamous cell carcinoma, HNSCC (85). Intriguingly, some years later the same group revealed cellular heparanase expression in late-stage HNSCCs was associated with prolonged overall survival and proposed that the proliferation-reducing effect of high heparanase levels might outweigh the tumor-promoting effects of heparanase in advanced tumors (86). Despite raising the importance of tumor type, stage, and role of heparanase in particular tumor microenvironment, other groups also found heparanase expression to be linked to poor prognosis. Heparanase expression is induced in HNSCC and is associated with tumors larger in size, increased invasiveness, and reduced patient survival (118). In cancer of the salivary glands, Vlodavsky and colleagues demonstrated very significant differences between those with high expressing heparanase tumors and those with low or no positive staining in terms of overall survival (66). Despite a lack of studies investigating the possible clinical correlates for Sulf1 or Sulf2 in HNSCC, there is evidence demonstrating that Sulf1 re-expression, which diminishes cell surface HSPG sulfation, interferes with both FGF2 and HGF signaling in SCCHN (33) and that desulfation of the cell surface HSPG by Sulf1 in SCCHN plays an important role in the control of tumor development (33).

### Breast and ovarian cancers

The role of heparanase and HS in breast cancer has been discussed comprehensively in a recent review (4) but emerging data continues to identify heparanase as a prognostic marker for tumor progression in breast cancer. For example, elevated heparanase expression was associated with the lymph node status, late clinical stages, a short overall survival, and a short relapse-free survival with the highest heparanase levels in breast cancer those with lymph node metastasis. In addition, the serum heparanase levels of patients with metastatic breast cancer were significantly higher than primary breast cancer patients (119). By contrast, Fernandez-Vega and colleagues did not find any significant differences between heparanase expression in tumors and healthy tissue. However, despite no significant difference in non-metastatic tumors, there was a change in metastatic relative to healthy tissue. Expression of heparanase determined in tissue arrays by immunohistochemistry revealed varying levels of heparanase in different patients (120). Nevertheless, laboratory studies now implicate heparanase in an invasive cell phenotype driven by the small GTPases: Rac1 and RhoA in brain metastatic breast cancer cells. These actions of heparanase on Rac1 and RhoA are mediated by GEF-H1, suggesting roles for heparanase in the initial events of BMBC pathogenesis, for example, cell adhesion, cytoskeletal dynamics, and cell extravasation, which are independent of its enzymatic activity (121). The clinical value of using heparanase, as a prognostic biomarker, in combination with MMP-9 and Cathepsin, was reported for determining the extent of ovarian cancer metastasis before surgery (81).

Both Sulf1 and Sulf2 were shown to be overexpressed at gene and protein levels in non-metastatic invasive ductal carcinomas although no significant differences could be detected in metastatic tumors (120). However, a recent review article highlighted the down regulation of Sulf1 in early stage ovarian tumors and metastatic breast cancer patients (7) while assessment of Sulf2 using a cohort of breast cancer patients found significant upregulation in autologous metastatic lesion compared with primary tumors (122). It is of interest to note that as intimated in the PDAC setting, the tumor type, the Sulf isoform (Sulf1 and/or Sulf2), and the predominant pathway (Wnt or FGF2) might result in opposing effects of Sulf’s on tumor progression and metastasis (122).

Sulf1 has been shown to be markedly downregulated in ovarian cancer cell lines and 75% of ovarian cancer tumor tissues (43, 123). In addition, a transcription factor vHNF has been shown to suppress Sulf1 expression in clear cell carcinoma (123), whereas HIF-1 alpha have been shown to suppress Sulf1 transcription in breast cancer cell lines (64). A subsequent study showed that restoration of Sulf1 expression leads to decreased tumor growth, angiogenesis, and enhanced the efficacy of chemotherapeutic agents such as cisplatin (124). Tumor suppressive effects of Sulf1 are predominantly linked to its ability to decrease FGF2, HB-EGF, amphiregulin signaling in ovarian and breast cancer cells (30, 64, 101). Decreased 6-*O* sulfation of heparan sulfate due to increased Sulf1 activities can also be recapitulated or mimicked by downregulation of 6-*O*-sulfotransferases (HS6ST1 and HS6ST2). More recently, it has been shown that downregulation of HS6ST1 reduced the signaling of HS-binding EGF signaling leading to reduced expression of FGF 1, IL6, and IL8 (125) in ovarian cancer cell lines. These data lend further support to the notion that decreased 6-*O* sulfation of HS results in reduced formation of the receptor-HS-growth factor ternary complex thereby limiting the effect of extracellular growth factors. Clinically, it has been shown that Sulf1 expression is associated with increased disease-free and overall survival in breast cancer (64). Similarly, analysis of 501 ovarian patients revealed that serous tumors with moderate to high levels of Sulf1 (127 of 186, 68%) showed a trend toward improved survival as assessed by Kaplan–Meier survival analysis and log-rank test (123). These data confirm an *in vitro* finding indicating a tumor suppressor role of Sulf1.

### Non-small cell lung cancer

Recent studies have identified the overexpression of heparanase in non-small cell lung cancer (NSCLC) (83, 126). Widespread Sulf2 protein expression was identified in tumor cells of 10/10 surgical specimens of human lung squamous carcinomas and knockdown of Sulf2 was found to reduce the growth of lung cancer cells, inhibit autocrine Wnt signaling, and inhibit tumor progression in a mouse model of NSCLC (127). The authors concluded that not only should Sulf2 be considered as a potential biomarker of lung cancer but that its inhibition could be achievable via small molecule or biologic agents and thus should be considered as a therapeutic target in lung cancer.

### Other solid tumors

For further examples of the studies that detail the clinical association studies with heparanase, readers are referred to several reviews (6, 74, 75). Similarly, comprehensive reviews on Sulf1 and Sulf2 provide further details of these enzymes in different cancer types (8, 76). In addition, an over-representation of Sulf2 gene expression in skin cancer, colorectal carcinoma, testicular teratoma, and liver cancer compared to their normal tissue counterpart has been reported (93). Further investigations revealed that Sulf2 was significantly overexpressed in high grade uveal melanoma compared to low grade and in patients presenting colorectal carcinoma compared to benign colon adenoma (93).

## Hematologic Tumors

### Myeloma

Of particular interest in the field is the role of heparanase in myeloma because its overexpression in the bone marrow environment was associated with a shorter event-free survival of patients with newly diagnosed myeloma treated with high-dose chemotherapy and stem cell transplantation (52). Heparanase enhances osteoclastogenesis and bone loss – a major cause of morbidity in patient with multiple myeloma – by shifting the differentiation potential of osteoblast progenitors from osteoclastogenesis to adipogenesis possibly via induction of the Wnt signaling pathway inhibitor DKK1 by both osteoblast progenitors and myeloma cells (128). Such effects on osteoclastogenesis and bone loss can occur, in part, as the result of a significant elevation in the expression and secretion of receptor activator of NF-κB ligand (RANKL) by heparanase-expressing myeloma cells. Another possible mechanism for the pro-tumorigenic effects of heparanase in myeloma was recently elucidated in animal models, which revealed that heparanase enhances myeloma progression via CXCL10 downregulation (129). In addition to an association of heparanase with myeloma progression, Bret and colleagues discovered that Sulf2 expression in primary multiple myeloma cells were associated with a poor prognosis in two independent large cohorts of patients. It remained an independent predictor when considered together with conventional multiple myeloma prognosis factors (93).

### Leukemia

In mononuclear cells derived from various leukemias, heparanase mRNA was expressed in 14 of 15 acute myeloid leukemia (AML) samples. In contrast, cells derived from all 33 chronic lymphoblastic leukemia, all 7 non-Hodgkin’s lymphoma, 7 of 8 chronic myeloid leukemia, and 6 of 8 acute lymphoblastic leukemia patients showed no detectable expression of the heparanase RNA. Heparanase protein was detected primarily within the cytoplasm of AML cells, indicating that the enzyme is produced and stored within the cytoplasm of myeloid cells, with limited expression on the cell surface (88). The low heparanase gene expression level in ALL patients and a high expression level in MM and AML patients were confirmed in a follow-up study (89). A clear correlation was found between heparanase mRNA expression level and three HPSE gene single nucleotide polymorphisms (SNPs – rs4693608, rs11099592, and rs4364254) among healthy individuals. These data suggested that certain HPSE gene SNPs contributes to basal heparanase gene expression and that alterations in this gene are an important determinant in the pathogenesis of hematological malignancies (89). Sulf1 gene expression has been noted to be increased in T prolymphocytic leukemia and AML (93).

## Therapeutic Opportunities

### Heparanase inhibitors

The development of heparanase inhibitors have been reviewed elsewhere (75, 130, 131) but Table 2 highlights the drug discovery and development projects initiated in the pursuit of heparanase inhibition and their current status. PI-88 is a mixture of highly sulfated oligosaccharides derived from *Pichia holstii*, NRRL Y-2448, which is a non-cleavable structural mimic of HS (132) and is currently progressing through clinical development as a dual anti-angiogenic and anti-metastatic agent. As such, it is known to inhibit angiogenesis by interfering with HS recognition by many angiogenic growth factors such as VEGF, FGF1, FGF2, in addition to inhibiting heparanase activity (133). Notably, PI-88 has been shown to inhibit primary tumor growth of invasive rat mammary adenocarcinoma, metastasis, and reduced vascularity of these tumors (134) while demonstrating significant anti-tumor activity in the pancreatic neuroendocrine RIP2-Tag2 model (135) and in models of leukemia (136). As the most advanced heparanase inhibitor, it is currently being trialed in a Phase III study as an adjuvant treatment for patients with hepatitis virus related hepatocellular carcinoma after surgical resection (137).

**Table 2:** **Past and present drug discovery/development programs targeting heparanase in oncology**.

Company	Compound	Development stage	ClinicalTrials.gov Identifier/Reference
Medigen Biotechnology Corporation (Taiwan)	Muparfostat (PI-88)	Phase III (current)	NCT01402908
Momenta Pharmaceuticals (US)	Necuparanib (M402)	Phase I/II (current)	NCT01621243
Sigma Tau Pomezia (Italy)	Roneparstat (SST0001)	Phase I (current)	NCT01764880
Progen Pharmaceuticals (Aus)	PG545 from PG500 series	Phase I (current)	NCT02042781
Oxford Glycosciences (UK)	OGT2115	Preclinical (discontinued)	(131)
Imclone Systems (US)	Compound 7a	Preclinical (discontinued)	(138)
InSight Biopharmaceuticals (Israel)	Compound 4	Preclinical (discontinued)	(139)
	Compounds 1, 6		(140)
	Compound 2		(141)
	Compound 3		(142)
Astra Zeneca (UK)	Antibodies	Late discovery (discontinued)	(143)
Endotis Pharma (France)	EP80061 from EP-8000 series	Discovery/preclinical (discontinued)	(14, 144)
Unknown (Shanghai Institute of Materia Medica, China)	JG3 (oligomannurarate sulfate)	Early preclinical	(145, 146)
RIKEN Discovery Research Institute (Japan)	RK-682	Discovery	(147)
	KI-105	Discovery	(148)

Heparan sulfate mimetics or glycol-split heparins are considered the most common therapeutic approach to create novel oncology agents with some others currently in early stage clinical development. PG545 is a synthetic, potent competitive inhibitor of heparanase (149) demonstrated to possess significant anti-tumor, anti-angiogenic, and anti-metastatic activity in a variety of animal models (150–152). This agent is currently being assessed in a Phase I trial for patients with advanced solid tumors (153). M402 is a rationally engineered, non-cytotoxic HS mimetic, designed to inhibit multiple factors implicated in tumor–host cell interactions, including VEGF, FGF2, SDF1α, P-selectin, and heparanase demonstrating anti-metastatic activity alone and in combination with cisplatin or docetaxel in the orthotropic 4T1 murine mammary carcinoma model (154). Necuparanib (M402) is current under investigation with nab-paclitaxel and gemcitabine in pancreatic cancer (155). Roneparstat (SST0001) is an *N*-acetylated, glycol-split heparin, which inhibits heparanase, downregulates HGF, VEGF, and MMP-9 expression and suppresses angiogenesis. Roneparstat also diminishes heparanase-induced shedding of syndecan-1, which is known to be a potent promoter of myeloma growth (156). Roneparstat is currently being tested in a Phase I trial for patients with advanced multiple myeloma (157). Oligommanurarate sulfate (JG3), a novel marine-derived oligosaccharide, was also reported as a heparanase inhibitor (145), but has yet to be progressed to the clinic (as defined by registration on www.clinicaltrials.gov).

Apart from HS mimetics, small molecule inhibitors, and neutralizing antibody programs were developed by various companies including Oxford Glycosciences (158, 159), Imclone Systems (138, 160), and InSight Biopharmaceuticals (139–142), but the candidates failed to reach clinical trials. Although the reasons for the limited progression through to clinical development are unknown, the lack of a crystal structure for the heparanase protein may have been one of the confounding issues. But, it is interesting to note that the induction of endoplasmic reticulum stress by chemotherapeutic reagents is involved in the enhanced invasion and migration ability of breast cancer cells and inhibition of heparanase (using one of these inhibitors, OGT2115) suppressed the invasion and migration ability of breast cancer cells. This provides a strong rationale for the development of heparanase-based therapeutics for the prevention of metastasis induced by chemotherapeutic reagents (161). The use of OGT2115 combined with cisplatin led to significant inhibition of cell proliferation, invasion, and migration of human nasopharyngeal cells, further suggesting that combination approaches with heparanase inhibitors may improve outcomes with existing chemotherapeutic regimens (162).

Recent discovery stage reports have identified new putative heparanase inhibitors but these continue to be classified as heparin derivatives or HS mimetics. TD4-143-1, which is a selectively sulfated tetrasaccharide containing unsubstituted glucosamine residues, which inhibited heparanase activity and suppressed invasion of breast cancer cells *in vitro* (163). Although not designed to specifically inhibit just heparanase, a newly synthesized hexasaccharide was considered to possess typical heparanase inhibition profile consistent with LMWHs and fondapariunx (164). Nevertheless, in both instances the anti-heparanase activity would be considered to be modest which perhaps limits the utility of such approaches in terms of preclinical or clinical development.

### Sulfatase modulators

In considering Sulf1 or Sulf2 as drug targets, the main consideration is the increasing evidence implicating the Sulfs in cancer and whether these are responsible for augmenting cancer cell growth or inhibiting it (31, 32, 37, 101, 165). The HS mimetic PI-88 was demonstrated to inhibit the Sulfs with IC_50_ values in the range of 1.2–2.6 μg/mL, which is comparable to that of heparanase (166). Although not specific for the Sulfs, the study suggested that inhibiting the Sulfs should be linked to an anti-tumor effect. However, it has been recently demonstrated that the loss of Sulf1 expression promotes tumorigenicity in ovarian cancer cells through regulating Bim expression (42). In gastric cancer cells, expression of Sulf1 significantly suppressed cellular proliferation possibly through abrogating the Hedgehog signaling pathway (117). In fact, the expression of Sulf1 mediated by adenovirus in liver carcinoma cells downregulates the activity of AKT and ERK signaling pathways, and inhibits HCC cell migration and proliferation, which makes it a candidate anti-tumor factor for cancer gene therapy (167). These data are corroborated by another recent study using microRNA miR-21 which suppressed Sulf1 and enhanced the activity of liver carcinoma cell proliferation and xenograft tumor growth in mouse models (73).

Conversely, silencing of Sulf2 expression in breast cancer cells attenuated ductal carcinoma *in situ* progression to invasive ductal carcinoma *in vivo* (168). Moreover, proteasomal inhibitors such as bortezomib abolished Sulf2 expression in multiple breast cancer cells (122). Consistent with these studies, a disulfonyl derivative of phenyl–tert–butyl nitrone (PBN) called OKN-007 was shown to inhibit Sulf2 activities in hepatocellular carcinoma cell lines although its activity against Sulf1 has not been assessed. OKN-007 effectively inhibited tumor growth in HCC derived tumor xenografts exhibiting Sulf2 expression (169). Taken together, these studies suggest that while inhibiting Sulf2 may exert anti-tumor activities, the modulation of Sulf1 may have a divergent impact on tumor progression and be highly dependent on the tumor type and the key signaling pathways (e.g., Wnt) characterized to promote cell proliferation and survival in these tumors. Thus, significant inroads need to be made in researching the Sulfs as an appropriate target in cancer and identifying the optimal approaches needed to modulate their expression and activity in cancer.

## Conclusion

The modification of HSPG in the tumor microenvironment modulates a variety of processes that are important in cancer growth and spread. Three enzymes – heparanase, Sulf1 and Sulf2 – play crucial roles in regulating HSPG functioning in this compartment and the up- and down-regulation of these proteins in a large variety of cancers demonstrates how important they are in promoting or repressing malignancy. While the involvement of heparanase in progressing tumors is well-documented, both at the mechanistic level and in clinical observations, the roles of the Sulfs are not so clear, particularly for Sulf1. This clarity is reflected in the more advanced stage of clinical development that anti-heparanase therapeutics has reached in comparison to Sulf inhibitors. Resolution of the pro- and anti-tumor properties of the Sulfs and how these relate to tumor-stromal relationships, primary and metastatic lesion interactions, and other aspects of tumor growth is required before the development of anti-Sulf therapeutics can proceed with confidence. Nevertheless, there is a substantial body of data describing the importance of heparanase, Sulf1, and Sulf2 in modulating HS functioning in cancer, which highlights the already well founded view that targeting the ability of tumors to modify their microenvironment in order to promote growth and spread represents a solid therapeutic development pathway.

## Conflict of Interest Statement

Keith Dredge and Edward Hammond are employees of Progen Pharmaceuticals and are actively involved in the development of one of the experimental agents discussed in this review, PG545. The other co-authors report no conflicts of interest.
